# Bovine tuberculosis in working foxhounds: lessons learned from a complex public health investigation

**DOI:** 10.1017/S0950268818002753

**Published:** 2018-10-09

**Authors:** Emily Phipps, Kate McPhedran, David Edwards, Katherine Russell, Catherine M. O'Connor, Danielle A. Gunn-Moore, Conor O'Halloran, Tony Roberts, Jill Morris

**Affiliations:** 1 Public Health England South East, Chilton, OX11 0RE, UK; 2 Public Health England East of England, Thetford, IP24 1JD, UK; 3 Public Health England, Emerging Infections and Zoonoses, London, NW9 5EQ, UK; 4 Roslin Institute, University of Edinburgh Royal Dick School of Veterinary Studies, Easter Bush, Midlothian, UK; 5 Animal and Plant Health Agency, Wallingford, Oxfordshire, UK

**Keywords:** Bovine TB, domestic pets, occupation-related infections, screening

## Abstract

In 2017, Public Health England South East Health Protection Team (HPT) were involved in the management of an outbreak of *Mycobacterium bovis* (the causative agent of bovine tuberculosis) in a pack of working foxhounds. This paper summarises the actions taken by the team in managing the public health aspects of the outbreak, and lessons learned to improve the management of future potential outbreaks. A literature search was conducted to identify relevant publications on *M. bovis*. Clinical notes from the Public Health England (PHE) health protection database were reviewed and key points extracted. Animal and public health stakeholders involved in the management of the situation provided further evidence through unstructured interviews and personal communications. The PHE South East team initially provided ‘inform and advise’ letters to human contacts whilst awaiting laboratory confirmation to identify the infectious agent. Once *M. bovis* had been confirmed in the hounds, an in-depth risk assessment was conducted, and contacts were stratified in to risk pools. Eleven out of 20 exposed persons with the greatest risk of exposure were recommended to attend TB screening and one tested positive, but had no evidence of active TB infection. The number of human contacts working with foxhound packs can be large and varied. HPTs should undertake a comprehensive risk assessment of all potential routes of exposure, involve all other relevant stakeholders from an early stage and undertake regular risk assessments. Current guidance should be revised to account for the unique risks to human health posed by exposure to infected working dogs.

## Introduction

Detected infections of dogs with *Mycobacterium bovis*, the causative agent of bovine tuberculosis (TB), are extremely rare, with only seven cases being reported to the Animal and Plant Health Agency (APHA) between 2004 and 2010 [1]. The risk of transmission of *M. bovis* from dogs to humans is also considered rare, with expert consensus concluding that dogs are a ‘spill over’ host and not a significant source of transmission [2]. However, a 2017 outbreak of *M. bovis* amongst a pack of working foxhounds in the South of England sparked considerable interest and concern from members of the public, media, veterinary and health professionals, and led to a co-ordinated response from Public Health England (PHE) Health Protection Teams (HPTs), the University of Edinburgh and the APHA [3–6].

This was the first recorded outbreak of *M. bovis* in working foxhounds in England. Although public health guidance on the management of *M. bovis* associated with animals in general is available, there is no specific guidance describing the management of the human health risks associated with an outbreak of *M. bovis* in working foxhounds and the complex exposures this context presents. Given the increased awareness of *M. bovis* infection associated with working dogs (including foxhounds as seen here or other farm dogs) amongst the veterinary community, it is likely that assessments of the risks to human health will be required again in the future [1, 2]. Such situations are likely to attract wider public interest due to concerns over the spread of bovine TB.

Management of this unique outbreak in working foxhounds required PHE to work collaboratively with veterinary stakeholders to identify exposed persons and limit the spread of *M. bovis* amongst both humans and animals. This paper summarises the actions taken by PHE and associated stakeholders in managing the public health aspects of this situation, and lessons that can be learned to improve the management of similar situations following notification of potential outbreaks of *M. bovis* in dogs or other working animals, especially in the non-household setting.

## Methods

A literature search was conducted on PubMed and CAB Abstracts to identify relevant publications on *M. bovis* in dogs ((dog OR pack OR foxhound OR hound) AND (TB OR tuberculosis OR mycobacterium), all papers available on databases with English language translation available). Additional papers of relevance to this case study not identified through the literature search were suggested by authors.

Clinical notes from the PHE health protection database were reviewed in relation to the situation. Unstructured interviews and team discussions were held with PHE, University of Edinburgh and APHA staff involved in the management of the situation to obtain richer detail of the management approach and lessons learned.

## Results

### Background epidemiology of *M. bovis* in the UK

Bovine TB is an important disease of cattle with a wide range of wild and domestic animal hosts [7, 8]. The causative agent, *M. bovis*, is a slow growing, aerobic bacterium that can cause infection of the lungs in humans, but can also affect any organ in the body [9]. Human infection with *M. bovis* typically occurs through ingestion, inhalation or contact with mucous membranes or skin abrasions of an infected animal [7]. The main sources of zoonotic transmission of *M. bovis* infection are the ingestion of unpasteurised milk and dairy products, or prolonged exposure to aerosolised bacilli excreted from the respiratory tract of diseased animals [7, 10]. Localised non-pulmonary *M. bovis* lesions can occur rarely through handling infected animals or carcases and theoretically through aerosolised exposure in abattoirs [10]. Human-to-human transmission is extremely rare [9]. In the UK, infection in humans with *M. bovis* is much less common than *M. tuberculosis*; between 2002 and 2014, there were 357 reported human cases of *M. bovis* compared with 96 887 cases of *M. tuberculosis* [9, 11]. It is thought that most cases of *M. bovis* infection in humans in the UK are likely due to reactivation of latent infection acquired before compulsory pasteurisation of milk and cattle TB control programmes, contact with a human TB case or infection acquired abroad [7, 9].

The incidence rate of *M. bovis* in cattle in England is one of the highest in Europe (11.0 new herd incidents per 100 herd-years at risk in 2017) [12]. The UK government has implemented stringent controls to minimise the spread of bovine TB, with some counties in the South West and South East of England being deemed High Risk Area (HRA) and separate adjacent buffer zone (Edge Area), and so cattle in these areas are subject to additional testing and surveillance [12]. Non-bovine farmed animals such as South American camelids (alpacas), sheep, goats, pigs and deer are also sporadically infected, usually through direct or indirect contact with cattle or badgers (the two maintenance hosts of the bacterium in the UK) [13, 14]. Infection of companion animals does occur, and there have been numerous reported cases of pets testing positive for *M. bovis* over the past few years, predominantly domestic cats [2, 12, 13, 15]. The majority of cases of dogs infected with *M. bovis* in the literature come from countries other than England and have identified risk factors such as being strays or having exposure to infected non-domestic animals [1, 15–17].

The potential for zoonotic transmission of *M. bovis* from companion animals to human contacts was confirmed in 2014 when cases of human *M. bovis* infection followed transmission from infected cats in Berkshire, England [16–18]. Lessons learned from the management of exposure to infected companion animals in 2014 justified a measured and co-ordinated public health response in order to protect human health in the 2017 incident involving working foxhounds.

### Initial risk assessment

The responsible HPT in the South East of England were initially made aware in early 2017 of the potential risk of zoonotic transmission of *M. bovis* by a private veterinarian with clinical suspicion of this diagnosis in working foxhounds cared for at the practice. A number of veterinary staff may have come into contact with infected hounds during their routine work. Key questions asked by the public health team in the initial assessment are summarised in Box 1, based on core principles of health protection practice and adaptation of existing general guidelines on the management of human health risks of TB [7, 19]. Before implementing public health actions, it is recommended to wait for laboratory confirmation [7]. Veterinary practice staff were therefore reassured and ‘inform and advise’ letters offered for distribution in the workplace, and the situation highlighted to the APHA in case of further action required.

Box 1. Initial assessment

1. What is the name of the hunt and where is it located?
2. How many animals are involved and how many have undergone testing?
3. What clinical signs did the hound(s) present with?
4. What type of testing has been undertaken (e.g. PCR/culture/interferon-*γ* release assay (IGRA))?
5. When are the results available to confirm the diagnosis?
6. Any at-risk groups in contact with the hounds, e.g. immunocompromised staff?
7. Any symptomatic staff or household members?

### Confirming the diagnosis

Primary concerns regarding the health of the kennel hounds were first raised in late 2016; a number of hounds had been euthanased on welfare grounds for reasons of deteriorating health within the preceding months. When a new hound started showing similar clinical signs (weight loss, lethargy, pyrexia, polyuria and polydipsia), it was euthanased and submitted for *post-mortem* examination. Gross renal pathology was confirmed histopathologically as granulomatous, and the presence of acid-fast bacilli with mycobacterial morphology was confirmed by the examination of Ziehl–Neelsen stained sections of diseased tissue.

This finding instigated a veterinary disease outbreak investigation by the University of Edinburgh, Biobest Laboratories and APHA [20]. Briefly, 164 hounds in the kennel were tested using an experimental interferon-*γ* release assay (IGRA) test at Biobest Laboratories and a serological assay originally developed for cervid species (Dual Path Platform VetTB test for cervids).

Of the 164 hounds tested, 85 (52%) were diagnosed as being test positive *as per* the prospective case definition set [20]. Test positive hounds and clinically unwell hounds were euthanased and *M. bovis* infection was confirmed by culture in 14 cases. The isolated organism was genotyped by the APHA confirming type 10a, with the first laboratory confirmation occurring in February 2017.

### Veterinary epidemiological investigation

Once infection with *M. bovis* 10a had been confirmed, evaluation of the risk to human health was undertaken (see ‘Identifying at-risk groups’ below). Simultaneously, an epidemiological investigation began to identify the risk pathways by which the hounds may have initially become infected. These are detailed in [20] and comprised of, in order of considered likelihood; (a) movement of infected hounds into the kennels, (b) feeding *M. bovis* infected fallen stock to the hounds, (c) exposure to infected livestock or wildlife during work, and/or (d) exposure to infected local wildlife at the kennels.

Qualitative assessment of each pathway was undertaken with the evidence available. Tracing of hound movements indicated that 21 hounds had been moved onto the affected premises within the 3 years prior to the diagnosis of the index case, of which 18 were moved in the preceding 18 months. Some of these hounds came from kennels that were located within a geographical region of the UK designated as a HRA for *M. bovis* incidence with respect to bovine infections. Furthermore, a number of these were located within the home – range of *M. bovis* 10a. It was therefore considered to be medium risk (and therefore most probable) that whilst at these kennels, hounds were fed fallen stock infected with *M. bovis* 10a and they were then the source of infection to the outbreak-kennel.

The remaining pathways were deemed to be possible sources of infection but of low risk because, (a) the outbreak-kennel was located within the Edge Area for *M. bovis* incidence, (b) it was out of the home range of *M. bovis* 10a, (c) carcases fed in the previous year had been traced and assessed as having a low likelihood of being infected with *M. bovis* after consideration of the TB history and the epidemiology of any current TB incidents on local farms at the time of the collection, and (d) there had been no locally identified wildlife infections with *M. bovis.*

### Identifying at-risk groups

Following confirmation of *M. bovis* infection in the working foxhounds, PHE undertook a formal risk assessment to identify potential routes of zoonotic transmission, understand levels of exposure and determine future public health actions, including possible screening of human contacts. Available guidance related to *M. bovis* in livestock was reviewed and tailored to this specific situation [7]. The key points of this risk assessment are summarised in Box 2, and involved collecting information from staff caring for the foxhounds, the local veterinary practice and the APHA. Again, this risk assessment was undertaken based on the key principles of health protection practice and adaptation of general guidance on the management of human health risks related to bovine TB [7, 19].

Box 2. Summary details of the public health risk assessment

Potential routes of exposure

1. Identify all persons who were in contact with the symptomatic foxhounds including temporary and previous staff members at the kennels and veterinary practice
2. Define the level of contact and activities undertaken, e.g. preparing food, grooming, cleaning environment, dressing wounds, undertaking invasive procedures
3. Whether anyone may have been bitten by potentially infected foxhounds

Risk of exposure

1. Level of exposure – total time spent with infected foxhounds by each person involved
2. Use of personal protective equipment (PPE) during contact – consistency and type worn
3. Whether any contact could have generated aerosols

Environmental factors

1. Environments in which contact takes place, e.g. kennels, household, vehicles and veterinary practice
2. Condition of kennels, e.g. cracked concrete and other porous surfaces
3. Ventilation and cleanliness of environments
4. Frequency and level of cleaning undertaken, e.g. sweeping, pressure washing, disinfecting

Veterinary assessment

1. List of veterinary practices caring for the working foxhound pack
2. Incidence of bovine TB in the surrounding area amongst bovine and non-bovine animals
3. Potential route of initial infection, e.g. contact with confirmed bovine cases or being fed potentially contaminated meat
4. Follow-up being undertaken by APHA
5. Any plans to test the rest of the working foxhound pack

### Communications strategy

A multi-agency incident control team (ICT) was convened to facilitate effective information gathering and to agree on suitable actions for all involved stakeholders. This collective approach, also involving specialist communication colleagues from all agencies, ensured that consistent messages were delivered, with the aim of reducing misinformation and managing perceptions of risk amongst the public [21].

The foxhounds were exercised on land and at events over several counties in the South East. Local HPTs across the region received calls from members of the public, veterinary and public health professionals enquiring about the pack and associated risks of infection. Calls to HPTs were recorded on the PHE health protection database (inFact Shipley Ltd © 2012). In order to facilitate co-ordination between teams and quick referencing, a unique identifier for the situation was created that could be linked to all incoming enquiries taken by the teams involved. The response was coordinated across several HPTs by the incident lead, and an email circulation list was used to keep the incident management team informed of all actions undertaken and updates on the situation. PHE and Defra communications teams were also involved at this point to provide advice on appropriate communications. Defra and veterinary colleagues experienced a higher volume of interview and statement requests than the public health teams, likely due to the high profile of fox hunting and bovine TB in the media.

### Screening and further public health actions

A ‘stone in the pond’ approach was adopted in determining which human individuals should initially undergo screening for TB [22]. Screening does not differentiate between *M. bovis* and *M. tuberculosis* infections, but does identify persons requiring further investigations and confirmatory testing. PHE identified close contacts with the highest risk of potential transmission for initial screening, and then planned to expand the screening pool to more casual contacts if the first round of screening suggested significant transmission had occurred.

The individuals identified for the first round of screening were those with the greatest degree of contact with infected foxhounds, and included persons who had conducted invasive procedures on symptomatic dogs (such as *post-mortem* examinations and surgical procedures) without appropriate personal protective equipment (PPE), and kennel workers involved with food preparation and cleaning of kennels using a pressure washer. These were hypothesised to be the highest risk exposures to potential sources of *M. bovis*, e.g. infected tissue, aerosolised fluids and potential contaminated fallen stock used as feed. The use of a pressure washer was of particular interest as the presence of kidney lesions in infected foxhounds [20] suggests that *M. bovis* bacilli could have been excreted in the hounds’ urine.

Persons in the initial screening group were referred to their local TB service for screening. All other identified potential contacts were sent an ‘inform and advise’ letter whilst results from the initial round of screening were awaited (see Box 3 for outline summary). In addition, HPT staff visited the veterinary practice to discuss and allay staff concerns related to *M. bovis* and their risk of acquiring TB.

Box 3. Key information for inform and advise letters

1. Reassure that the risk of transmission from animals to humans is low
2. Some persons may be unusually susceptible such as immunocompromised persons
3. Description of the symptoms of TB
4. Advice to contact GP if experiencing any of these symptoms, mentioning the possible route of exposure through contact with the foxhounds
5. Screening of high-risk persons is currently taking place and we may be in contact again to arrange screening if initial tests indicate that transmission of bovine TB has occurred

**Fig. 1.**
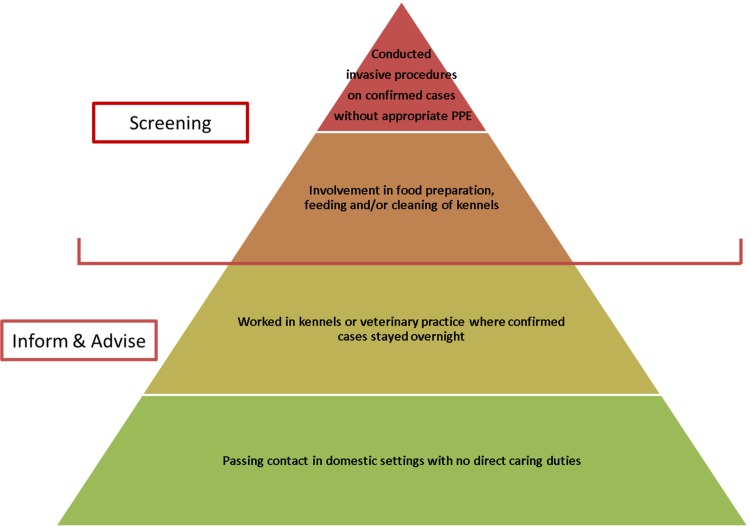
Hierarchy of screening pools.

In total, 11 out of 17 people were considered to have potential exposure to infected foxhounds as outlined in Fig. 1 and were offered screening for active and latent TB through IGRA testing and chest x-rays. Of these, one person tested positive for TB (using QuantiFERON^®^ TB Gold). Following a further assessment including CT scanning and culture of tissue samples, there was no evidence of active TB infection and latent TB was diagnosed. This person had not had previous testing for TB, did not have other risk factors for TB, but was involved in all high-risk activities including *post-mortem* examination without PPE, preparation of fallen carcases for feeding, cleaning of kennels using a pressure washer and care of open wounds on infected animals. Seven contacts screened negative for TB, and three declined the offer of screening. Based on this outcome of the first screening round, it was agreed by the ICT that further screening would not be offered to more casual contacts.

Screening of the foxhounds continued over several months with two further screening rounds. Hounds with a complete set of negative tests were allowed to move to a clean kennel and remain clinically healthy at the time of writing [20]. All foxhounds testing IGRA-positive were euthanised. A second meeting of the original multi-stakeholder ICT was convened to discuss how to manage the ongoing exposure to IGRA-positive hounds since the initial diagnosis. Staff members who had declined screening initially were encouraged to attend given this ongoing exposure. Three new staff members (bringing the total number of risk assessed persons up to twenty) who had joined the hunt since the initial diagnosis were identified and they were provided with ‘inform and advise’ letters to highlight their potential risk of exposure to infected foxhounds during both rounds of animal screening, and recommended actions if they develop symptoms indicative of potential TB infection.

## Discussion

The management of this situation has highlighted several learning points for veterinary and public health stakeholders. Outbreaks of potentially zoonotic infections amongst working foxhound packs are a unique challenge for both HPTs and veterinary investigation teams, as they are comprised of large populations (in this case over 150 hounds), which are highly mobile (this kennel covered an area spanning six counties) and hounds are legislatively defined neither as companion animals nor agricultural livestock, meaning, for example, that they can be fed fallen stock. The hounds from working packs may span both categories, spending time in both domestic and farming settings. This makes quantifying the unique exposures and risk of transmission associated with this context particularly difficult as they are not specifically covered by any existing guidelines. Moreover, IGRA testing is not validated for use in diagnosing *M. bovis* infections in dogs, so the sensitivity and specificity of any results are unknown, adding some uncertainty to whether public health action should be warranted in light of a positive test (R. Dempsey (2017) Personal communication). In this scenario, given the high number of positive IGRA tests, confirmatory tissue cultures and foxhounds with clinical signs consistent with TB, the decision to take action was relatively straightforward, but could be difficult in the face of less conclusive results.

Bovine TB is an understandably emotive subject for many stakeholders, including kennel staff, farmers, veterinarians and the general public, and there was a considerable media interest. It was crucial to ensure that confidentiality of the working foxhound pack was protected as much as possible by agencies involved, and that information sharing activities were coordinated and agreed on by all parties. The geographical area covered by the pack during outings spanned several counties, and required the involvement of multiple HPTs and TB services to coordinate a response. Effective communication between these teams is extremely important to ensure that resources are used efficiently and messages are consistent in order to address public concern. By visiting the veterinary practice in person, the HPT were able to build relationships and foster trust from stakeholders to manage concern successfully. This also presented an opportunity for highlighting the importance of wearing appropriate PPE when performing *post-mortem* examinations of animals, which would have significantly reduced the risk of exposure to *M. bovis* to veterinary and kennel staff.

This situation highlighted how dynamic risk assessments should be undertaken frequently during the ongoing process of outbreak management, as the foxhound pack is likely to come in to contact with new persons such as temporary staff and members of the public as well as those potential contacts identified in the initial assessment. IGRA testing of the whole pack was a significant undertaking for the kennel and University of Edinburgh staff, who were unable to secure additional funding for testing as the foxhounds were deemed to be non-livestock species, and took several months to complete. During this period, workers continued to be exposed to hounds that were eventually found to be IGRA-positive. Managing this unknown and unquantifiable risk of potential exposure whilst waiting for IGRA results was particularly challenging, and involved concerted effort from the HPT to communicate effectively with stakeholders to address and allay concerns. Senior managers of the foxhound kennels were very receptive to both public health requirements and advice regarding animal health management. It is worth noting that as the hounds are not classed as livestock, the APHA have no regulatory powers to enforce euthanasia or other control methods.

## Conclusion

This paper highlights the unique and unusual health protection scenario of managing potential working foxhound to human transmission of *M. bovis*. The number of human contacts with working packs can be large and varied, and the animals are not considered as domestic companion animals or livestock. HPTs involved in the management of such situations must ensure to undertake a comprehensive risk assessment of all potential routes of exposure, involve all other relevant stakeholders from an early stage in developing management and communication plans, and undertake regular risk assessments as new information becomes available. Current guidance should be revised to account for the unique risks to human health posed by exposure to infected working dogs.
